# Change in Housing Status among Homeless and Formerly Homeless Individuals in Quebec, Canada: A Profile Study

**DOI:** 10.3390/ijerph17176254

**Published:** 2020-08-27

**Authors:** Gesthika Kaltsidis, Guy Grenier, Zhirong Cao, Marie-Josée Fleury

**Affiliations:** 1 Department of Psychiatry, McGill University, Montreal, QC H3A 1A1, Canada; gesthimani.kaltsidis@mail.mcgill.ca; 2 Douglas Hospital Research Centre, Douglas Mental Health University Institute, Montreal, QC H4H 1R3, Canada; guy.grenier@douglas.mcgill.ca (G.G.); Zhirong.Cao@douglas.mcgill.ca (Z.C.)

**Keywords:** homelessness, type of housing, housing status, factors, typology, cluster analysis

## Abstract

Housing stability is a key outcome in studies evaluating housing services for the homeless population. Housing stability has typically been defined dichotomously and based on a fixed duration of maintenance in housing accommodations, which does not fully capture change in housing status among homeless individuals. Moreover, few typologies have examined housing trajectories across different housing types. Cluster analysis was used to develop a typology of housing status change for 270 currently or formerly homeless individuals in Quebec (Canada) residing in shelters and temporary and permanent housing. Participants were interviewed at baseline (T0) and 12 months later (T1). The Gelberg–Andersen Model was used to organize housing-related variables into predisposing, needs and enabling factors. Comparison analyses were conducted to assess group differences. Three groups (Groups 1, 3 and 4) had more favorable and two (Groups 2 and 5) less favorable, housing status at T1. Findings suggest that maintenance or improvement of housing status requires suitable types and frequencies of service use (enabling factors) that are well adapted to the nature and complexity of health problems (needs factors) among homeless individuals. Specific interventions, such as outreach programs and case management, should be prioritized for individuals at higher risk for returning to homelessness.

## 1. Introduction

Homelessness and housing instability have serious impact on the health and the well-being of individuals [1]. Enabling homeless individuals to access and maintain permanent housing (PH) is therefore an essential factor in their recovery [1]. Housing stability is a key outcome in studies evaluating housing services for the homeless population [2,3,4]. Recent systematic reviews have highlighted the lack of consensus in definitions of housing stability [5,6]. Most studies categorize participants as either housed or homeless [7,8,9] and include relatively few dimensions in defining housing trajectories, such as type of accommodation (e.g., living with family or friends, in supportive housing, etc.) and housing duration (e.g., 90 consecutive days, or longer) [5,6]. However, restricting the definition of housing stability to time-limited duration of housing maintenance fails to capture the housing trajectories of homeless individuals that range along a continuum of short-, medium- and long-term services, from emergency shelters and temporary housing (TH, i.e., housing offering accommodation during a period usually for up to 24 months) [10] to PH. Moreover, considering the heterogeneity among homeless population, it is likely that various characteristics may relate to housing outcomes: whether the housing status of previously homeless individuals may improve, remain stable or deteriorate over time.

One way to identify housing trajectories (or change in housing status) among homeless individuals is by typology. Typological research may reveal similar characteristics among subgroups of homeless individuals, facilitating the implementation of housing and other services that address their specific needs. Some studies have attempted to classify homeless individuals within a broad population [2,11] or in subpopulations such as veterans [12,13] and youth [14,15,16]. Several typologies have been established based on previous life experience [17,18], physical or mental health problems [13,19,20], quality of life [21,22] and patterns of emergency shelter use [23]. Although some recent studies have identified typologies of housing trajectories or outcomes [2,20,24], no study to our knowledge has identified profiles of homeless individuals based on their trajectories across various types of housing accommodation (e.g., emergency shelter to TH or TH to PH). Examining changes in housing status among individuals living in different housing conditions, as well their specific socio-demographic, clinical and service use characteristics, may contribute to a better understanding of housing stability.

Cluster analyses with homeless individuals have been conducted using multiple variables: sociodemographic (e.g., age and sex), clinical (e.g., mental health disorders (MHD) and substance use disorders (SUD)) and service use (e.g., frequency of emergency department visits (ED) and hospitalizations) [2,17,20,25]. Some typologies have included risk factors (e.g., victimization and arrest history) and protective factors (e.g., social support and positive perceived health) as pertinent variables [15,22]. However, several variables have been less studied with respect to housing stability, including suicidal behavior and functional disability, both very prevalent in homelessness [26]; use of public primary care services, such as having a family doctor [27,28]; or required codes of living/conduct in different housing models [29,30], for example enforcing stringent abstinence policies against substance use as opposed to the harm reduction policies characteristic of Housing First, a PH model with case management [31,32], which offers direct access for homeless individuals with serious MHD and/or SUD to a PH without the obligation to participate in treatment [33].

Despite the wide range of variables used to distinguish classes of homeless individuals, few studies have used a conceptual framework. Bonin et al. (2009) developed a typology of homeless individuals with MHD using the Network-Episode Model [19], which considers individuals entering the healthcare system in social context and within support networks [34]. Another widely used model in health service evaluation is the Gelberg–Andersen Behavioral Model for Vulnerable Populations [35], in which variables are classified as predisposing factors (i.e., socio-demographics: e.g., age and sex), needs factors (i.e., clinical variables: types and numbers of disorders) and enabling factors (i.e., service use variables, including having a family doctor or frequency of hospitalization) [35]. Previous studies of homelessness using the Gelberg–Andersen Model have identified predictors of outcomes, such as satisfaction [27], exit from supported housing [36], and health service use [37]. However, to our knowledge, no typology exists for variables organized within the Gelberg–Andersen Model.

The objective of this study was to develop a typology for housing status change using an adapted version of the Gelberg–Andersen Model, for a cohort of 270 currently or formerly homeless individuals residing in different types of housing in Quebec (Canada). A typology of homelessness based on changes in housing status may contribute to current understanding of housing stability among homeless or formerly homeless individuals and should inform housing policies and services that address the specific needs of identified subgroups.

## 2. Materials and Methods

### 2.1. Study Setting and Data Collection

This multisite study was conducted in Quebec’s two largest urban centers. Participants were recruited from 27 community or public organizations, 20 of which provided housing services. Five of them were emergency shelters; fourteen offered TH and three PH. Most TH involved 3–12-month residency. The remaining seven organizations offered other ancillary services: food banks, day centers, soup kitchens, etc.

Study participants had to be at least 18 years old, currently living in a shelter or TH or be previously homeless and living in HF within the past two years. In total, 497 individuals were invited to participate in the study: 46 emergency shelter users, 243 TH residents and 208 PH residents. While no individuals interested in the study who met eligibility criteria were excluded, interviews were sometimes postponed to accommodate anyone intoxicated or otherwise unfit to be interviewed. Recruitment strategies included: posters displayed in common areas of the selected organizations, on-site recruitment by the project coordinator and referrals by housing staff who attended information meetings given by project researchers.

Interviews were administered by trained research assistants, closely supervised by the research team. Baseline interviews were managed from January to September 2017 (T0), usually the same day or shortly after initial contact. Follow-up data collection for participants interviewed at T0 occurred approximately 12 months later, between January and December 2018 (T1). T0 interviews usually lasted 75 min and T1 interviews were typically shorter, about 55 min, since some sociodemographic items remained unchanged in the 12-month interlude. Interviews were conducted at the selected organizations, participant apartments or local restaurants. Participants completed a questionnaire consisting of questions on socio-demographics (e.g., age and education); residential history (e.g., chronic homelessness); service use, including satisfaction with services (e.g., having a family doctor and emergency department use); and diagnoses (e.g., MHD, SUD and physical illness). Prior to the interview, each study participant provided written informed consent. After completing interviews, participants received a modest financial compensation for their time and contribution to the study. 

Program coordinators in the participating housing resources (*n* = 47) also completed a short questionnaire concerning support programs they provided. This questionnaire included 14 items related to the nature of their code of living/conduct protocols for residents (e.g., alcohol or drug use and participation in community activities). This questionnaire was self-administered, available online through LimeSurvey software or was conducted in person by a trained research assistant between November 2017 and March 2018. The multisite study protocol was approved by the research ethics board of the Douglas Mental Health University Institute (IUSMD 16/35).

### 2.2. Conceptual Framework, Variables and Instruments

**Figure 1:**
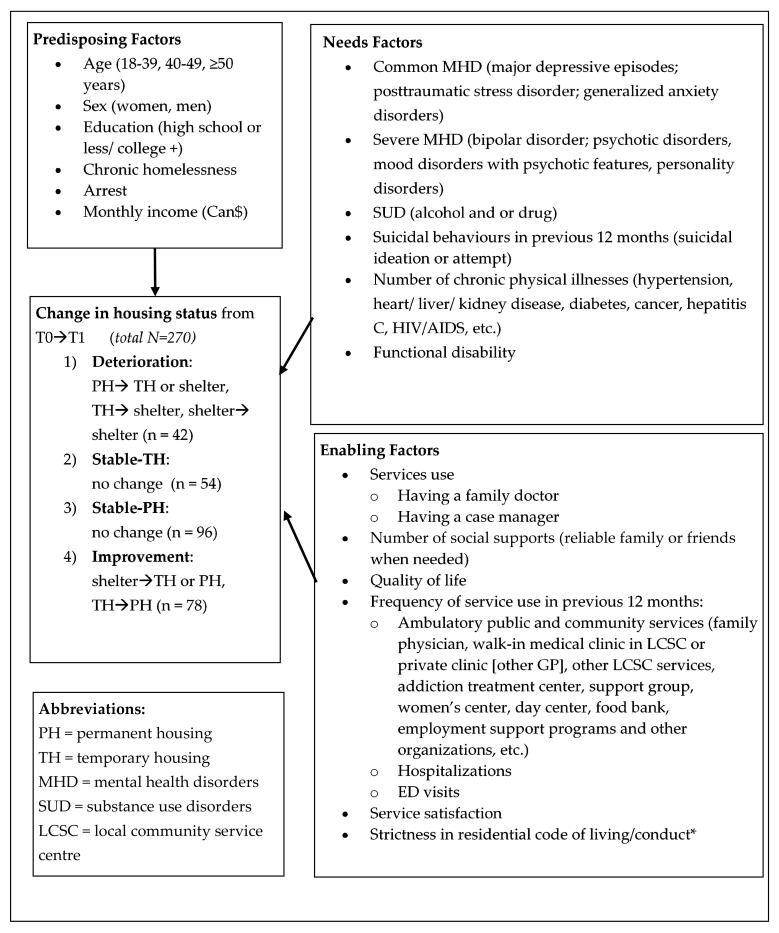
Conceptual framework for change in housing status based on the Gelberg–Andersen Behavioral Model for Vulnerable populations.

All study variables were based on the Gelberg–Andersen Model [35], as presented in Figure 1. Standardized instruments [38,39,40,41,42,43,44,45] used for both T0 and T1 interviews are listed in Table S1. The main variable of interest for the basis of the cluster analysis was change in housing status from T0 (baseline) to T1 (12 months). Housing status at both T0 and T1 was determined using an adapted version of the Canadian Community Health Survey (CCHS) where participants reported one of three accommodation types: emergency shelters (overnight stays less than a week), TH (3–12 month residency) and PH (with apartment lease of usually 12 months renewable). Participants were then grouped into the following four conditions related to the change in housing status at T1: (1) deterioration (PH to TH or shelter, TH to shelter, shelter to shelter by T1); (2) stable-TH (no change); (3) stable-PH (no change); and (4) improvement (shelter to TH or PH, TH to PH).

Independent variables were identified based on their relevance to the homelessness literature and organized into predisposing, needs and enabling factors according to the Gelberg–Andersen Model [35]. Predisposing factors included: age, sex, education, chronic homelessness, arrest (for theft, violence, drugs, etc.) and monthly income. Chronic homelessness was defined as a single homeless episode of at least 12 months or 4 homeless episodes within a 3-year period [46]. Needs factors for the previous 12 months included: common MHD (e.g., major depressive episodes and anxiety disorders), severe MHD (e.g., bipolar disorder, psychotic disorders and personality disorders), SUD (alcohol and/or drug), suicidal behavior, number of chronic physical illnesses (e.g., hypertension and kidney disease) and functional disability. Enabling factors referred to: having a family doctor or case manager, number of social supports (reliable family or friends when needed), quality of life, frequency of service use in the previous 12 months (ambulatory public and community services, hospitalizations and ED visits), service satisfaction score and strictness in residential code of living/conduct.

### 2.3. Analysis

Univariate analyses with all independent variables from T1 consisted of frequency distributions for categorical variables and mean values with standard deviations for continuous variables. Missing values (less than 5%) were randomly distributed and imputed by expectation maximization method [47]. Cluster analysis was conducted using the k-means group algorithm with Gower dissimilarity coefficient, and several k-means solutions with different numbers (3–7) of groups were computed [48]. The five-group solution had the largest Calinski–Harabasz pseudo-F value, indicating that the five-group solution was most distinct as compared with the other groups. Comparison analyses were conducted to assess statistical differences between groups: Chi-square or Fisher’s exact tests were used for categorical variables, and T-tests or the Wilcoxon rank-sum test for continuous variables. Stata version 15 was used to conduct the group analyses.

## 3. Results

Of the 497 participants recruited, 455 enrolled at baseline (T0, 92%) and 270 at 12-month follow-up (T1), for a response rate of 59% at T1. Comparative analyses using cross tabulations on categorical variable showed no differences in gender between the T0 and T1 samples (*p* = 0.518). T-test was used for the continuous variables, age and disability, at T0 and T1 yielding no significant differences (age: *p* = 0.126; disability: *p* = 0.677). No significant differences were found between individuals lost to follow-up (*n* = 185) and those retained (*n* = 270), in terms of baseline characteristics for sex (*p* = 0.199), education (*p* = 0.689) and disability (*p* = 0.330). In addition, of 47 program coordinators contacted to participate in the study, 44 completed the questionnaire (79% women; mean age 42 years) for a response rate of 94%.

Among the 270 participants at T1, 96 (36%) were in stable-PH and 54 (20%) in stable-TH, while change in housing status reflected improvement for 78 (29%) and deterioration for 42 (16%). Among the 78 participants whose housing status had improved, 72 (98%) moved from TH to PH, 4 (1.5%) from emergency shelters to PH and 2 (0.5%) from emergency shelter to TH. Among the 42 participants whose housing status had deteriorated at T1, 20 (48%) moved from PH to TH, 11 (26%) used emergency shelters throughout the 12-month period, 8 (19%) moved from TH to emergency shelters and 3 (7%) from PH to emergency shelters.

Participant characteristics at T1 are presented in Table 1. Regarding predisposing factors, 57% were 50 years old or over, 58% were men and 67% had high school education or less. About half (52%) had experienced chronic homelessness; 16% were arrested in the previous 12 months; and average monthly income was $959.34. Concerning needs factors, 72% of participants reported severe MHD or personality disorders, 43% common MHD, 37% SUD and 24% suicidal behavior. Thirty-five percent of participants had at least one chronic physical illness for an average of 0.60 chronic physical illnesses. Participants had a mean score of 21 on the 60-point disability scale, indicating moderately compromised functionality. Regarding enabling factors, 57% reported having a family doctor, and 50% a case manager. Participants received social support from an average of two people, and mean quality of life score was 71 on a 100-point scale. In the previous 12 months, participants reported using ambulatory public and community services 88 times, visited the ED twice and were hospitalized 0.50 times on average. Mean service satisfaction score was 4.0/5.0. Strictness in residential code of living/conduct was assessed as 8.7/14.

Cluster analysis identified five groups related to change in housing status at T1 (Table 2). Detailed group comparison tests for each variable are shown in Table S2. Three groups (Groups 1, 4 and 3) had more favorable housing conditions at T1. Group 1 was the largest, representing 25% of the sample (*n* = 68/270) with 59% of participants showing improved housing status at T1 and 41% in stable-PH. Group 4 represented 23% of the sample (*n* = 61/270), 57% of whom showed improved housing status by T1 and 43% remained in stable-PH. Group 3 accounted for 17% of the sample (*n* = 47/270) with 81% in stable-PH, while 13% experienced housing status deterioration and 6% housing status improvement at T1. The two remaining groups (Groups 2 and 5) had less favorable housing status at T1. Group 2 included 20% of the sample (*n* = 54/270) with half in stable-TH, 43% experiencing housing status deterioration and 7% in stable-PH. Finally, Group 5, the smallest group, included 15% of the sample (*n* = 40/270) with 68% in stable-TH and 33% experiencing housing status deterioration by T1.

Group profiles are described below in order of more favorable change in housing status over 12 months (Groups 1, 4 and 3), followed by those in less favorable housing status (Groups 2 and 5). Group 1 differed significantly from Groups 2 and 5 (less stable-PH) as well as Group 3 (more stable-PH). All Group 1 individuals were 50 years old or older and had low risk of suicidal behavior, differing from those in Groups 3–5. Men and women were represented almost equally in Group 1, unlike Groups 2 and 4. Disability and service satisfaction scores were more favorable for Group 1 than Groups 3 and 4. Group 1 participants were less affected by common or severe MHD or personality disorders and used fewer ambulatory public and community services than those in Group 3. Group 1 had fewer arrests than Group 5 and lived in housing with more strict codes of living or conduct than those in housing where Group 2 and 3 participants lived. Group 1 was labeled: “Older individuals with fewer MHD, less disability and fewer arrests, residing in stable-PH or experiencing housing status improvement.”

Group 4 participants all experienced housing status improvement or lived in stable-PH and consisted mainly of women. Group 4 had more individuals in their 40s and fewer with chronic physical illnesses relative to Groups 1–3. Group 4 participants reported more suicidal behavior and higher disability scores than in Group 1. Social support was higher in Group 4, as was the number of hospitalizations and ED visits relative to Group 2. The Group 4 service satisfaction score was lower than those for Groups 1 and 2, and the score for strictness of residential code of living/conduct was higher than scores for Groups 2 and 3. Group 4 was labeled: “Middle-age women with high social support, few chronic physical illnesses, residing in stable-PH or experiencing housing status improvement at T1, but elevated disability and risk for suicidal behavior, high frequencies of hospitalization and ED visits.”

Group 3 individuals were in their 40s or older and lived predominantly in stable-PH. Their housing had the lowest scores for strictness in residential code of living/conduct. They made greater use of ambulatory services than Groups 1, 2 and 5, but had more ED visits than Group 2 only. Service satisfaction score for Group 3 was lower than those of Groups 1 and 2. The proportion of women in Group 3 was higher than Group 2, but lower than Group 4. Suicidal behavior was more prevalent in Group 3 compared with Groups 1 and 2. Group 3 participants were more likely to be affected by common or severe MHD or personality disorders and had higher disability scores compared with Group 1. Group 3 was labeled: “Middle-age to older individuals with high health needs and service use, residing mainly in stable-PH, and whose residences had lower strictness in residential codes of living/conduct.”

Group 2 had more men, and worse housing status at T1 compared with Groups 1, 3 and 4; half lived in stable-TH. All Group 2 individuals were 50 years of age or older. Group 2 registered a higher number of chronic physical illnesses than Groups 4 and 5. Both suicidal behavior and ambulatory service use were reported less in Group 2 than in Group 3. Strictness in residential code of living/conduct was low, differing from all other groups, while service satisfaction score was higher than in those for Groups 3 and 4. Group 2 participants also made fewer ED visits than did Groups 3 and 4 and fewer hospitalizations than Group 4. Participants were more likely to have a family doctor than those in Group 5 and had lower number of social supports than in Group 4. Group 2 was labeled: “Older men with poorer physical health, housing status deterioration at T1 or stable-TH, but having a family doctor and using fewer services.”

Group 5 participants were all living in stable-TH or had experienced housing status deterioration at T1, which differed from results for Groups 1, 3 and 4. Group 5 individuals were men mainly in their 40s. The average monthly income for Group 5 was the lowest of the five groups. More Group 5 participants were arrested than in Group 1. They had fewer chronic physical illnesses and were less likely to have a family doctor than Group 2 participants. They used fewer ambulatory services than Group 3 and experienced more strictness in residential code of living/conduct compared with Groups 2 and 3. Group 5 was labeled: “Middle-age men with low income and low ambulatory service use, more previous arrests, residing in stable-TH or experiencing housing status deterioration at T1.”

## 4. Discussion

This study established a typology for current or recently homeless individuals in Quebec based on change in housing status and across different housing types within a 12-month follow-up period. The great majority of participants maintained stable-PH or improved housing status by T1, which is comparable to results for HF studies reporting positive residential stability outcomes, even extending beyond 12 months [4,6,20]. Furthermore, few participants had experienced a deterioration of their housing status, representing a very positive result, which confirms the importance to offer to most homeless individuals a PH, as recommended in programs like Housing First.

Five groups were identified through cluster analysis, three (Groups 1, 3 and 4) showing more favorable housing status at T1 (stable-PH or improvement) and two (Groups 2 and 5) demonstrating less favorable housing status (stable-TH or deterioration) over the same period. Some groups showed similarities to those described in previous studies. For instance, studies identified a profile of homeless individuals living mainly in stable PH but having complex mental and physical health problems [2,20], similar to Group 3 in the present study. Adair et al. [20] also identified a group of homeless individuals with little housing stability, but relatively high monthly income and a high level of psychiatric symptoms, similar to the present Group 2. Another group mainly consisting of men with low monthly incomes and poor outcomes was quite similar to our Group 5 [20]. This latter group was probably the one that best represented the typical profile of homeless individuals as imagined by the general public. However, this group was the less numerous among the five identified by the cluster analysis. Bonin et al. [19] also identified a class of mainly women, with considerable social support and high service use, quite similar to Group 4. In contrast to previous studies [2,49], we did not identify a group with few health problems, the closest being Group 1 in this study. In addition, we were unable to identify a group mainly affected by SUD, unlike previous studies [2,21].

The results reveal notable differences between groups in terms of predisposing, needs and enabling factors. Concerning predisposing factors, Groups 2 and 5 showed less favorable housing status and were predominantly men, while women were more numerous in groups with more favorable housing status. Previous studies have demonstrated links between female gender and housing stability [20,50]. No other predisposing, needs or enabling factors were solely associated with change in housing status.

Several differences characterized the three groups registering change to more favorable housing status. Groups 1 and 4 had similar distributions in housing status, with all participants demonstrating stable-PH or housing status improvement after 12 months despite very different profiles. Concerning predisposing factors, Group 1 individuals were 50 years of age or older, whereas those in Group 4 were more in their 40s. Concerning needs factors, the fewest chronic physical illnesses among Group 4 may have reflected their relatively younger age. Group 4 also had significantly more suicidal behavior and was more affected by functional disability. Associations between functional disability and suicide risk have been previously reported [51,52]. In terms of enabling factors, Group 1 participants used few ambulatory public and community services, but reported higher service satisfaction than did Groups 3 and 4, suggesting that their health needs may have been met despite lower frequency of health and social service use [53]. By contrast, the higher prevalence of individuals with suicidal behavior in Group 4 may explain their more frequent hospitalizations and ED use [54]. Moreover, the preponderance of women might explain greater social support in Group 4, as women tend to benefit from larger networks of family and friends [55,56]. Some studies have reported positive associations between social support and housing stability [7,50,57].

Group 3 had a rather distinct profile especially on enabling and needs factors. Concerning enabling factors, these participants had the lowest score on strictness in residential code of living/conduct, which seems logical, given that over 80% of Group 3 individuals lived in stable-PH, which generally does not impose strict sets of rules on residents regarding abstinence from alcohol or drugs, as compared with residential codes in emergency shelters or TH. PH generally allows greater personal autonomy and provides a more agreeable living environment [29]. Moreover, Group 3 participants used more ambulatory public and community services yet had the lowest service satisfaction score. The combination of high service use and low satisfaction was likely due to greater prevalence of health problems (common and severe MHD, personality disorders, suicidal behavior and chronic physical illnesses) as well as in the highest level of functional disability. High health-related needs usually drive people to seek services more frequently. Moreover, Group 3 was the only group where most participants reported having a case manager (a perquisite in HF) and/or a family doctor, which facilitates health service use according to previous studies [58,59]. However, multiple health problems may lead to greater service dissatisfaction when services lack continuity or quality of care [60,61].

Groups 2 and 5, featuring individuals with less favorable housing status showed notable differences from participants in Groups 1, 3 and 4. Regarding predisposing factors, Group 5, predominantly male, reported the lowest monthly income and highest proportion of individuals arrested over the study period. As previously confirmed, being male [20,62], with a history of arrest [57,63] and/or low income [7,62], was associated with poorer housing stability. Concerning needs factors, the high prevalence of chronic physical illnesses reported by Group 2 was probably related to older age, physical health issues and associated frailty [26]. Despite the favorable housing status enjoyed by participants in Groups 1 and 3, a similar association was noted due to their numbers of older individuals with physical health problems, as compared with the younger Group 4. Regarding enabling factors, the main difference between Groups 2 and 5 was the proportion of individuals having a family doctor, lowest in Group 5 and highest in Group 2. The larger proportion of individuals having a family doctor in Group 2 may explain their lower frequency of ED visits and hospitalizations as well as their high service satisfaction score, with the family doctor acting as a protective factor [27,28]. However, Group 2 participants and those from Group 5 used few ambulatory public and community services, depriving them of valuable assistance in addressing their mental health problems. This may also have contributed to their housing status deterioration at T1. This underutilization of ambulatory public and community services may have resulted from the fact that less than 50% of participants of Groups 2 and 5 had a case manager. Furthermore, Group 2 was overrepresented by men who are usually least endowed with social support [55,56]. Finally, a surprising finding was that strictness in residential code of living/conduct for Group 2 was significantly lower than that of Groups 1 and 4, suggesting that some TH where Group 2 participants were living had fairly relaxed codes of living/conduct.

Some limitations were present in this study. First, convenience sampling was used which implies that the sample may not be representative of the homeless population. Second, there were considerably fewer young people (under age 40) than middle-age or older people (age 40 and over) among participants. Better profiling of younger individuals experiencing homelessness would provide insight into their specific needs [26,57]. Third, as we have no information on the residential status of individuals who did not participate in T1, it is impossible to know if the profiles reported here were representative of the study’s baseline (T0) sample. Fourth, as data were collected in Quebec, our results are specific to this area and may not be generalized, particularly to jurisdictions without universal health insurance [64]. Finally, the study relied on self-report data. For certain variables (e.g., arrest history, MHD and frequency of service use), clinical records could have been used to verify and complement information provided by participants.

## 5. Conclusions

This was the first known study to develop a typology based on change in housing status over 12 months for homeless individuals residing in three different types of accommodations. Findings revealed five groups, of which three groups (Groups 1, 3 and 4) showed more favorable and two groups (Groups 2 and 5) experienced less favorable change in housing status over 12 months. The study considered variables tested in relation to housing trajectory, some of them less studied or novel, such as suicidal behavior, chronic physical illnesses, functional disability, having a family doctor, service satisfaction and strictness in residential code of living/conduct. The results show that maintaining or improving housing status may be attained by homeless individuals with various profiles. The key element in housing status improvement or maintenance seems related to the type and frequency of service use (enabling factors) that need to be well adapted to the nature and complexity of health problems (needs factors) of the homeless population. Moreover, specific interventions adapted to the diverse profiles of this population are suggested. Considering Groups 2 and 5, which were those experiencing the worst deterioration in housing status and were mainly constituted of men, outreach programs or case management could be prioritized, with a view toward increasing access to services and better meeting their specific needs. For Group 2, mainly constituted of older men with chronic physical illnesses, the services of a nurse making home visits could also be helpful. For Group 5, programs facilitating access to work would be appropriate in order to increase this group income. For Groups 3 and 4, which were mainly individuals in stable housing but with high health needs, adequate use of primary and specialized care services is essential to prevent a decline in their physical and mental health conditions that could put them at risk of returning to homelessness. The services of specialized educators may be useful especially for Group 3 because of their high functional disability. Concerning Group 4, a greater utilization of case managers may be necessary due to the high frequency of ED visits among these participants. Finally, for Group 1, a case manager and when needed of their family doctor are probably sufficient to satisfy their needs.

## Figures and Tables

**Table 1:** Participant characteristics at T1 (12-month follow-up; *n* = 270).

Variables	Min	Max	*n*/Mean	%/SD.
			270	100.00
Sample size				
**Change in Housing Status**				
Deterioration			42	15.56
Stable-TH			54	20.00
Stable-PH			96	35.56
Improvement			78	28.89
**Predisposing Factors**				
Age (mean/SD)	19	76	49.52	11.23
18–39 years			14	5.19
40–49 years			103	38.15
50 and over			153	56.67
Sex				
Women			114	42.22
Men			156	57.78
Education				
High school or less			182	67.41
College or more			88	32.59
Chronic homelessness			141	52.22
Arrest			44	16.3
Monthly income (CAD; mean/SD)	0	8880	959.34	756.98
**Needs Factors**				
Common MHD			117	43.33
Severe MHD including personality disorders			194	71.85
Substance use disorders			101	37.41
Suicidal behaviors			64	23.7
Number of chronic physical illnesses (mean/SD)	0	5	0.60	0.98
Functional disability ^1^ (mean/SD)	11	46	20.64	6.58
**Enabling Factors**				
Having a family doctor			153	56.67
Having a case manager			136	50.37
Number of social supports (mean/SD)	0	13	2.18	2.44
Quality of life ^2^ (mean/SD)	35	100	71.05	9.76
Frequency of ambulatory public and community service use ^3^ (mean/SD)	0	632	87.55	120.67
Hospitalizations (mean/SD)	0	7	0.50	1.11
ED visits (mean/SD)	0	100	1.98	8.38
Service satisfaction ^4^ (mean/SD)	1	5	3.99	0.76
Strictness in residential code of living/conduct ^5^ (mean/SD)	0	14	8.66	4.92

TH, temporary housing; PH, permanent housing; MHD, mental health disorders; ED, emergency department. ^1^ Functional disability: score with rating: 0–60 where 0 = no disability and 60 = full disability. ^2^ Quality of life: score with rating: 20–100 where higher = better quality of life. ^3^ Ambulatory public and community services: general practitioners (both family doctors or any doctors in walk-in clinics), first line biopsychosocial services in local community service centers (LCSC), ambulatory care in hospitals, addiction treatment centers, support group, women’s center, day center, food bank, employment support programs, etc.). ^4^ Service satisfaction: score based on previous 12 months where higher = more satisfied with services. ^5^ Strictness in residential code of living/conduct: score completed by program coordinators at housing services organizations where higher = stricter program rules for living/conduct.

**Table 2:** Cluster analysis of change in housing status over 12 months.

Variables	Group 1		Group 2		Group 3		Group 4		Group 5	
	*n*/Mean	%/SD	*n*/Mean	%/SD	*n*/Mean	%/SD	*n*/Mean	%/SD	*n*/Mean	%/SD
Group size	68	25.19	54	20.00	47	17.41	61	22.59	40	14.81
**Change in Housing Status**										
Deterioration	0	0.00	23 ^1,3,4^	42.59	6 ^1,2,4,5^	12.77	0	0.00	13 ^1,3,4^	32.50
Stable-TH	0	0.00	27	50.00	0	0.00	0	0.00	27	67.50
Stable-PH	28 ^2,3,5^	41.18	4	7.41	38	80.85	26 ^2,3,5^	42.62	0	0.00
Improvement	40	58.82	0	0.00	3	6.38	35	57.38	0	0.00
**Predisposing Factors**										
Age										
18–39 years	0	0.00	0	0.00	0	0.00	9	14.75	5	12.50
40–49 years	0	0.00	0	0.00	16	34.04	52 ^1,2,3^	85.25	35 ^1,2,3^	87.50
50 and over	68 ^3,4,5^	100	54 ^3,4,5^	100	31 ^1,2,4,5^	65.96	0	0.00	0	0.00
Female	31 ^2,4^	45.59	10 ^1,3,4^	18.52	18 ^2,4^	38.30	44 ^1,2,3,5^	72.13	11 ^4^	27.50
Education (college or more)	22	32.35	18	33.33	12	25.53	22	36.07	14	35.00
Chronic homelessness	33	48.53	32	59.26	26	55.32	29	47.54	21	52.50
Arrest	6 ^5^	8.82	8	14.81	10	21.28	10	16.39	10 ^1^	25.00
Monthly income (CAD; mean/SD)	917.11 ^5^	347.34	1257.64 ^5^	1534.39	851.28 ^5^	188.94	958.45 ^5^	420.43	756.74 ^1,2,3,4^	221.43
**Needs Factors**										
Common MHD	26 ^3^	38.24	21	38.89	27 ^1^	57.45	25	40.98	18	45.00
Severe MHD including personality disorders	41 ^3^	60.29	39	72.22	39 ^1^	82.98	46	75.41	29	72.5
Substance use disorders	23	33.82	23	42.59	18	38.3	20	32.79	17	42.5
Suicidal behaviors	7 ^3,4,5^	10.29	10 ^3^	18.52	18 ^1,2^	38.3	19 ^1^	31.15	10 ^1^	25.00
Number of chronic physical illnesses (mean/SD)	0.63 ^4^	0.96	0.98 ^4,5^	1.12	0.70 ^4^	1.12	0.25 ^1,2,3^	0.62	0.45 ^2^	0.90
Median (IQR)	0.00	(0,1)	1.00	(0.2)	0.00	(0.1)	0.00	(0.0)	0.00	(0.1)
Functional disability (mean/SD)	18.85 ^3,4^	4.45	20.46	7.45	22.77 ^1^	7.98	21.06 ^1^	6.04	20.77	6.82
**Enabling Factors**										
Having a family doctor	42	61.76	37 ^5^	68.52	26	55.32	30	49.18	18 ^2^	45.00
Having a case manager	33	48.53	25	46.3	30	63.83	30	49.18	18	45.00
Number of social supports (mean/SD)	2.34	2.64	1.54 ^4^	2.18	1.98	1.81	2.56 ^2^	2.47	2.45	2.89
Quality of life (mean/SD)	71.21	8.96	72.89	11.32	71.98	11.03	69.57	8.48	69.45	8.92
Frequency of ambulatory public and community service use (mean/SD)	68.50 ^3^	102.27	87.02 ^3^	134.63	120.00 ^1,2,5^	128.00	98.13	133.62	66.38 ^3^	92.18
Median (IQR)	29.00	(5,63)	19.00	(5.98)	66.00	(23.205)	43.00	(8.150)	34.00	(10.81)
Hospitalizations (mean/SD)	0.54	1.25	0.28 ^4^	0.71	0.36	0.85	0.66 ^2^	1.12	0.63	1.46
Median (IQR)	0	(0,1)	0	(0.0)	0	(0.0)	0	(0.1)	0	(0.1)
ED visits (mean/SD)	2.53	10.74	0.76 ^3,4^	1.66	1.40 ^2^	2.19	3.28 ^2^	12.97	1.38	3.54
Median (IQR)	0	(0,2)	0	(0.1)	1	(0.2)	1	(0.2)	0	(0.1)
Service satisfaction score (mean/SD)	4.13 ^3,4^	0.63	4.18 ^3,4^	0.75	3.80 ^1,2^	0.93	3.88 ^1,2^	0.66	3.92	0.82
Strictness in residential code of living/conduct (mean/SD)	11.04 ^2,3^	1.75	9.53 ^1,3,4,5^	3.92	0.13 ^1,2,4,5^	0.88	11.29 ^2,3^	1.58	11.56 ^2,3^	2.38

TH, temporary housing; PH, permanent housing; MHD, mental health disorders; ED, emergency department, Superscript numbers indicate significant differences at *p* < 0.05, Profiles—Group 1: “Older individuals with fewer MHD, less disability and fewer arrests, residing in stable-PH or experiencing housing status improvement.” Group 2: “Older men with poorer physical health, housing status deterioration at T1 or stable-TH, but having a family doctor and using fewer services.” Group 3: “Middle-age to older individuals with high health needs and service use, residing mainly in stable-PH, and whose residences had lower strictness in residential codes of living/conduct.” Group 4: “Middle-age women with high social support, few chronic physical illnesses, residing in stable-PH or experiencing housing status improvement at T1, but elevated disability and risk for suicidal behavior, high frequencies of hospitalization and ED visits.” Group 5: “Middle-age men with low income and low ambulatory service use, more previous arrests, residing in stable-TH or experiencing housing status deterioration at T1.”.

## Supplementary Materials

The following are available online at https://www.mdpi.com/1660-4601/17/17/6254/s1, Table S1: Variables and instruments based on the Gelberg-Andersen Behavioral Model, Table S2: Comparison tests between groups (*p*-values).
