# Supplementary material for: Change in Housing Status among Homeless and Formerly Homeless Individuals in Quebec, Canada: A Profile Study
Source: Int J Environ Res Public Health. 2020 Aug 27;17(17):6254. doi: 10.3390/ijerph17176254 (PMC7504688; doi:10.3390/ijerph17176254)
Supplement: Supplementary file 1 [file ijerph-17-06254-s001.pdf]

Table S1. Variables and instruments based on the Gelberg-Andersen Behavioral Model [35].

	Instruments & References	Description	Psychometric Properties
**Main variable of interest**			
Housing status (categorical)	Canadian Community Health Survey (CCHS)--adapted[38]	Self-report; three types of housing (emergency shelter, temporary housing, permanent housing with or without support)	N/A
**Predisposing factors**			
Age (categorical)	CCHS--adapted [38]	Self-report; numerical value calculated from date of birth	N/A
Sex (categorical)		Self-report; two-point scale (male = 1, female = 2)	
Education (categorical)		Self-report; two-point scale (high school or less = 1, college or more = 2)	
Chronic homelessness (categorical)		Self-report; *chronic homelessness* refers to a single homeless episode of at least 12 months, or 4 homeless episodes within a 3-year period [46]; two-point scale (no = 0; yes = 1)	
Arrest (categorical)		Self-report; *arrest* includes theft, violence, drugs, etc. in the past 12 months, two-point scale (no = 0; yes = 1)	
Monthly income (continuous)		Self-report; in Canadian $	
**Need factors**			
Mental health disorders (MHD) (categorical)	M.I.N.I International Neuropsychiatric Interview 6.0 [39]	120-item structured diagnostic interview for DSM-IV and ICD-10 psychiatric disorders; two-point scale (no = 0; yes = 1)	Kappa Cohen = 0.50-0.84
Personality disorders (categorical)	Standardized Assessment of Personality Abbreviated Scale [40]	8-item semi-structured interview from Standardized Assessment of Personality; two-point scale (no = 0; yes = 1)	Cronbach's alpha = 0.68
Substance use disorders (SUD) (categorical)	Alcohol Use Disorders Identification Test (AUDIT) [41]	10-item self-report scale measuring alcohol consumption; with zero to four-point scoring for multiple-choice questions; rating: 0–50 where higher = greater level of SUD for alcohol	Cronbach's alpha = 0.74
	Drug Abuse Screening Test (DAST) [42]	28-item self-report scale used as a screening tool for drug consumption; two-point scale (no = 0; yes = 1); rating: 0–20 where higher = greater level of SUD for drugs	Cronbach's alpha = 0.88
Suicidal behaviors (categorical)	CCHS)--adapted [38]	Self-report; *suicidal behaviors* refer to suicidal ideation or attempt; two-point scale (no = 0; yes = 1)	N/A
Number of chronic physical illnesses (continuous)		Self-report; *CPI* include hypertension, heart/ liver/ kidney disease, diabetes, cancer, hepatitis C, HIV/AIDS etc.; numerical value	
Functional disability (continuous)	WHO Disability Assessment Schedule 2.0 [43]	12-item short version assessment used for all diseases (physical illnesses and MHD); across 6 domains of functioning (cognitive, mobility, self-care, getting along, life activities, and participation); five-point scale (1 to 5); rating: 0 to 60 where 0 = no disability and 60 = full disability	Cronbach's alpha = 0.93-0.94
**Enabling factors**			
Having a family doctor (categorical)	CCHS--adapted [38]	Self-report; two-point scale (no = 0; yes = 1)	N/A

Having a case manager (categorical)		Self-report; two-point scale (no = 0; yes = 1)	
Number of social supports (continuous)		Self-report; *social support* refers to family or friends that one can rely on when in need; numerical value	
Quality of life (QOL) (continuous)	Satisfaction with Life Domains Scale (SLDS) [44]	20-item self-report scale on subjective QOL; across 5 domains (daily life and social relations, housing and neighborhood, personal relationships, spare-time activities, autonomy); five-point scale (1 to 5); rating: 20 to 100 where higher = better QOL	Cronbach's alpha = 0.92
Frequency of ambulatory public & community service use (continuous)	Service Utilization Questionnaire (SUQ) adapted from CCHS [45]	Self-report; *Ambulatory public and community services* refer to a wide range of health and social services; numerical value based on previous 12 months	N/A
Hospitalizations (continuous)		Self-report; numerical value based on previous 12 months	
Emergency department visits (continuous)			
Service satisfaction (continuous)		Self-report; Overall satisfaction with services; five-point scale (1 to 5) based on previous 12 months where higher = more satisfied with services	
Strictness in residential code of living/conduct (continuous)	Questionnaire for organizations offering housing services	14-item evaluation on organization's strictness in residential code of living/conduct; completed by program coordinators at housing service organizations; two-point scale (no = 0; yes = 1) where higher = stricter program rules for living/conduct	N/A

Table S2. Comparison tests between groups (*p*-values).

	Group 1 vs 2	Group 1 vs 3	Group 1 vs 4	Group 1 vs 5	Group 2 vs 3	Group 2 vs 4	Group 2 vs 5	Group 3 vs 4	Group 3 vs 5	Group 4 vs 5
**Main variable of interest**										
Change in housing status[1]	**<0.0001*****	**<0.0001*****	0.868	**<0.0001*****	**<0.0001*****	**<0.0001*****	0.091	**<0.0001*****	**<0.0001*****	**<0.0001*****
**Predisposing factors**										
Age[1]	**---**	**<0.0001*****	**<0.0001*****	**<0.0001*****	**<0.0001*****	**<0.0001*****	**<0.0001*****	**<0.0001*****	**<0.0001*****	0.748
Female	**0.002****	0.437	**0.002****	0.063	**0.027***	**<0.0001*****	0.301	**<0.0001*****	0.287	**<0.0001*****
Education	0.909	0.431	0.657	0.778	0.392	0.759	0.866	0.243	0.336	0.913
Chronic homelessness	0.238	0.474	0.911	0.690	0.690	0.209	0.514	0.423	0.793	0.626
Arrest	0.302	0.058	0.193	**0.022***	0.397	0.816	0.215	0.517	0.681	0.288
Monthly income (Can$)	0.078	0.239	0.542	**0.010***	0.074	0.146	**0.044***	0.107	**0.034***	**0.006****
**Need factors**										
Common MHD	0.941	**0.042***	0.750	0.490	0.062	0.819	0.552	0.090	0.247	0.690
Severe MHD (+ personality disorders)	0.168	**0.009****	0.067	0.200	0.199	0.698	0.976	0.341	0.238	0.744
Substance use disorders	0.321	0.622	0.901	0.367	0.661	0.278	0.993	0.552	0.690	0.322

Suicidal behaviors	0.193	**<0.0001*****	**0.003****	**0.043***	**0.027***	0.120	0.448	0.438	0.186	0.504
Number of chronic physical illnesses[2]	0.074	0.964	**0.004****	0.200	0.133	**<0.0001*****	**0.010***	**0.008****	0.237	0.182
Functional disability	0.140	**0.001****	**0.019***	0.078	0.137	0.638	0.837	0.207	0.218	0.826
**Enabling factors**										
Having a family doctor	0.438	0.489	0.151	0.090	0.172	0.036	**0.022***	0.527	0.337	0.681
Having a case manager	0.806	0.105	0.941	0.723	0.078	0.757	0.901	0.129	0.078	0.681
Number of social supports	0.075	0.419	0.629	0.838	0.275	**0.021***	0.084	0.180	0.358	0.842
Quality of life	0.361	0.680	0.291	0.327	0.684	0.076	0.115	0.203	0.248	0.944
Frequency of ambulatory public & community service use[2]	0.998	**0.002****	0.164	0.520	**0.006****	0.265	0.553	0.080	**0.018***	0.550
Hospitalizations[2]	0.328	0.469	0.203	0.804	0.841	**0.026***	0.272	0.072	0.379	0.369
ED visits[2]	0.090	0.622	0.467	0.323	**0.037***	**0.025***	0.614	0.799	0.165	0.132
Service satisfaction	0.683	**0.028***	**0.031***	0.147	**0.028***	**0.025***	0.120	0.615	0.532	0.776
Strictness in residential code of living/ conduct	**0.010***	**<0.0001*****	0.450	0.229	**<0.0001*****	**0.006****	**0.007****	**<0.0001*****	**<0.0001*****	0.548

MHD: mental health disorders; ED: emergency department; Significance between groups at ****p*<0.0001; ***p*<0.01; **p*<0.05; [1] Fisher's exact test; [2] Wilcoxon rank-sum test.
